# Tryptophan-Kynurenine Pathway in COVID-19-Dependent Musculoskeletal Pathology: A Minireview

**DOI:** 10.1155/2021/2911578

**Published:** 2021-10-05

**Authors:** Sagar Vyavahare, Sandeep Kumar, Nicholas Cantu, Ravindra Kolhe, Wendy B. Bollag, Meghan E. McGee-Lawrence, William D. Hill, Mark W. Hamrick, Carlos M. Isales, Sadanand Fulzele

**Affiliations:** ^1^Department of Cell Biology and Anatomy, Augusta University, Augusta, GA, USA; ^2^Department of Medicine, Augusta University, Augusta, GA, USA; ^3^Department of Pathology, Augusta University, Augusta, GA, USA; ^4^Department of Physiology, Augusta University, Augusta, GA, USA; ^5^Center for Healthy Aging, Augusta University, Augusta, GA, USA; ^6^Department of Pathology, Medical University of South Carolina, Charleston, SC, USA

## Abstract

Severe acute respiratory syndrome coronavirus 2 (SARS-CoV-2) causes coronavirus disease 2019 (COVID-19), affecting multiple organ systems, including the respiratory tract and lungs. Several studies have reported that the tryptophan-kynurenine pathway is altered in COVID-19 patients. The tryptophan-kynurenine pathway plays a vital role in regulating inflammation, metabolism, immune responses, and musculoskeletal system biology. In this minireview, we surmise the effects of the kynurenine pathway in COVID-19 patients and how this pathway might impact muscle and bone biology.

## 1. Introduction

**Figure 1:**
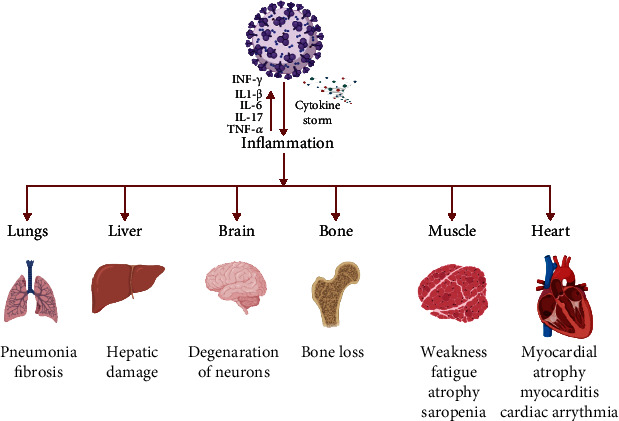
Illustration of impact of COVID-19 caused by infection with SARS-CoV-2 on various human organs-lungs, liver, brain, bone, muscle, and heart. (Figure is created by using http://BioRinder.com.)

Severe acute respiratory syndrome coronavirus 2 (SARS-CoV-2) is responsible for the current pandemic, suspected to originate from infected bats [1]. Coronavirus disease 2019 (COVID-19), caused by SARS-CoV-2, has turned out to be a major global catastrophe affecting millions of individuals across the globe [2]. In the United States, as of today, more than 30 million lives have been affected by COVID-19, and over six hundred thousand Americans have lost their lives, according to the Johns Hopkins Coronavirus Resource Center [3]. COVID-19 can present a wide spectrum of symptoms such as cough, fever, shortness of breath, muscle pain, and loss of taste and smell [4]. Mild to severely affected patients may experience elevated proinflammatory cytokines such as IL-1, TNF- *α*, and IL-6 [5], which negatively affect human health (Figure 1). Excessive activation of these proinflammatory cytokines (cytokine storm) leads to the alteration of several metabolic signaling pathways (e.g., the tryptophan-kynurenine pathway).

Recent studies have shown that the tryptophan-kynurenine pathway (Trp-Kyn) is altered in COVID-19 patients. A study conducted by Thomas et al. analyzed serum metabolites of COVID-19 patients and found that tryptophan (Trp) levels were reduced, and L-kynurenine (Kyn) was elevated [5]. A study performed by Fraser et al. reported similar findings (elevated levels of Kyn in COVID-19 patients) [6]. Another study reported that Kyn levels were elevated, along with kynurenic acid (Kyn-A) and quinolinic acid (QA) in the serum of COVID-19 patients [7]. The study conducted by Lawler et al. demonstrated elevated levels of QA in the blood plasma of COVID-19 patients [8]. Sex-specific differences have also been reported in the levels of Kyn-A and QA metabolites in COVID-19 patients. Serum metabolic analyses performed by Cai et al. reported elevated levels of Kyn-A in male patients compared to female patients [9]. Lionetto et al. assessed serum metabolites in COVID-19 patients and found that Kyn/Trp levels were elevated in male patients [10]. Moreover, Cai et al. (2020) reported an elevated Kyn-A: L-Kyn was associated with increased severity of COVID-19 infection in male patients [9]. The studies mentioned above indicate that activation of the tryptophan-kynurenine pathway might be one of the reasons for the increased susceptibility of males to COVID-19 infection.

Several studies also reported elevated levels of genes involved in tryptophan metabolic pathways [11, 12]. The study conducted by Policard et al. reported that indoleamine-pyrrole 2,3-dioxygenase (IDO-1) is significantly upregulated in COVID-19 patients [11]. Another study also reported similar findings showing elevated levels of IDO-1 in COVID-19 patients [12]. The study conducted by Grunewald et al. in the murine model demonstrated that IDO-1, IDO-2, and TDO-2 were significantly upregulated in murine coronavirus infection [13]. The prevalence and severity of COVID-19 disease are directly associated with age and the underlying condition, such as diabetes, obesity, and cardiovascular disorders [14, 15]. It is well known that the tryptophan-kynurenine pathway elevated with age and above mentioned underlying conditions [16].

The findings from these studies strongly indicate that the Trp-Kyn pathway is altered in COVID-19 patients, leading to a decrease in Trp levels and an increase in Kyn and its metabolites. Recent studies also demonstrated reduced muscle mass and bone loss in COVID-19 patients [17–20]. Based on the findings from our group and published literature, we came up with a novel perspective suggesting that the activation of the Trp-Kyn pathway in COVID-19 patients might be involved in bone and muscle loss.

## 2. The Tryptophan-Kynurenine (Trp-Kyn) Pathway

Tryptophan (Trp) is an essential amino acid that plays a vital role in protein synthesis, growth, mental health, and immune responses [21]. As age advances, proinflammatory cytokines, such as IL-6, IL-1*β*, and IFN-*γ*, lead to the activation of indoleamine 2,3-dioxygenase (IDO-1) [22]. An increase in levels/activity of IDO-1 along with inflammaging further leads to immunosuppression, neurodegenerative disorders, cardiovascular diseases, and fragility [21–24]. Augmentation of the levels/activity of IDO-1 decreases Trp levels and leads to the generation of several Trp intermediate metabolites [25]. Trp is catabolized by rate-limiting enzymes such as indoleamine 2,3-dioxygenase-1 (IDO-1), indoleamine 2,3-dioxygenase-2 (IDO-2), and tryptophan 2,3-dioxygenase-2 (TDO-2) into N-formylkynurenine and Kyn [26]. Further, Kyn is broken down into Kyn-A and 3-hydroxykynurenine by kynurenine aminotransferases (KAT) and kynurenine 3-monooxygenase (KMO) [27]. Trp also acts as a substrate for the generation of nicotinamide adenine dinucleotide (NAD+) through the conversion of quinolinic acid. NAD+ plays a crucial role in regulating several cellular processes, including energy production, chromosome stability, immune cell signaling, longevity mechanisms, and DNA repair [28, 29]. The Kyn and its metabolites induce downstream signaling by directly activating Ahr signaling [30] and/or indirect activation of the MEK- (mitogen-activated protein kinase (MAPK)/extracellular signal-regulated kinase (ERK) kinase-) ERK1/2 MAPK signaling pathway [31, 32].

**Figure 2:**
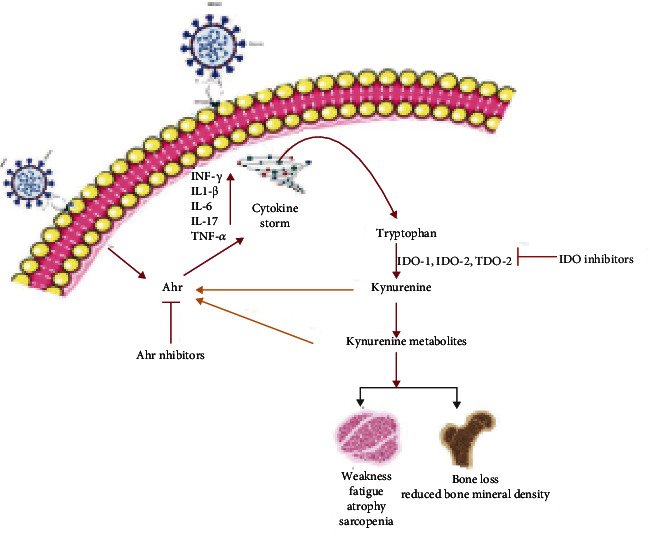
Overview of effects of SARS-CoV-2 infection on the muscle and bone. The SARS-CoV-2 infection elicits systemic inflammation (Cytokine storm), which activates the tryptophan-kynurenine pathway. Kynurenine is broken down into several downstream metabolites, which further activates AhR signaling, affecting the integrity and structure of the musculoskeletal system. (Figure is created by using http://BioRinder.com.)

IDO-1 is a master regulator of the Kyn pathway and downstream regulator of interferon signaling [33], which is activated during viral infection [34]. On the other hand, it has been reported that interferon-*γ* stimulates the expression of ACE2 (the receptor for SARS-CoV-2) in COVID-19 infection [35]. Hence, the interferon-*γ* signaling cascade potentiates inflammation in SARS-CoV-2 pathology [5]. Enhanced inflammation further leads to an increase in IDO-1 activity followed by enhanced degradation of Trp into Kyn and its metabolites. Our group identified the Trp-Kyn catabolic pathway as a novel causal mechanism in age-associated musculoskeletal complications (stem cell dysfunction and muscle and bone loss). We hypothesized that elevated levels of Kyn and its metabolites might be involved in COVID-19 musculoskeletal pathophysiology (Figure 2).

## 3. The Try-Kyn Pathway in COVID-19-Induced Musculoskeletal Pathophysiology

Kyn is known to increase with age and is involved in deleterious effects on the musculoskeletal system [24, 36–38]. Recently published data have demonstrated a loss of bone and muscle in COVID-19 patients [17–20]. We hypothesize that an increase in cytokine levels leads to activation of the IDO-Kyn pathway, which raises the levels of Kyn and its metabolites, leading to activation of the aryl hydrocarbon receptor (AhR) and downstream signaling. Induction of AhR signaling directly by viral particles [39] or by Kyn metabolites leads to bone and muscle loss. Viral infection activates AhR through an IDO1-AhR-IDO1-positive feedback loop, which eventually causes upregulation of downstream effectors, such as TCDD-inducible PARP (TiPARP), and enhances the expression of cytokines (e.g., interleukin IL-1*β*, IL-10, and TNF-*α*) [39]. Therefore, we hypothesize that elevations in the cytokine expression elicit IDO-Kyn-AhR activation that results in bone and muscle loss.

There is conclusive evidence demonstrating that Kyn increases bone resorption by activating the AhR signaling pathway [38, 40, 41]. An increase in Kyn levels accelerates skeletal aging, leading to decreased osteoblast numbers and increased osteoclast numbers and activity, resulting in bone loss via decreased formation and enhanced resorption [42]. The study performed by our group analyzed the direct effects of feeding Kyn on bone mass and also evaluated the short-term effects of intraperitoneal injection of Kyn on bone turnover in CD-1 mice [24]. Micro-CT analysis revealed a significant bone loss upon Kyn feeding in adult mice, and serum analysis revealed an increase in the levels of osteoclastogenic markers such as RANKL and pyridinoline crosslinks (PYD) [24]. Our study also reported an increase in bone marrow adiposity with Kyn treatment. Moreover, bone marrow stromal cells isolated from Kyn-injected mice showed a decrease in the expression of Hdac-3 and its cofactor NcoR1 and augmentation of the expression of lipid storage genes such as Cidec and Plin1 [24], suggesting a phenotype similar to accelerated aging since such changes are also observed in aged bone marrow cells [43]. A study conducted by Kalaska et al. revealed that elevated Kyn levels decrease bone strength in rats [44]. Kyn metabolites may also exert effects on bone: a study performed by Darlington et al. measured the ratio of 3-hydroxyanthranilic acid to anthranilic acid and found that anthranilic acid levels were increased, and 3-hydroxyanthranilic acid levels were decreased in osteoporotic patients [45].

Studies performed by our group have shown that in vitro treatment of RAW264.7 cells, a macrophage-like cells line, with Kyn induces osteoclastogenesis by upregulating osteoclast transcription factors (such as c-fos and NFATc1) which leads to an increase in TRAP+ osteoclasts [40]. Another metabolite, Kyn-A, inhibits the differentiation of osteoblasts and increases osteoclastogenesis through the extracellular signal-regulated kinase (ERK) pathway [36, 46]. Another study conducted by our group demonstrated that Kyn treatment of human and mouse myoblasts increases reactive oxygen species formation [47]. Consistent with this in vitro studies, in vivo treatment of mice with Kyn leads to increased lipid peroxidation accompanied by reduced muscle size and muscle strength [47]. Several Trp downstream metabolites such as Kyn, Kyn-A, and 3-hydroxykynurenine are endogenous AhR ligands likely to induce musculoskeletal damage [38, 40, 41, 48].

The decline in tryptophan levels and elevated levels of Kyn and its metabolites postcovid will affect not only musculoskeletal health but also accelerate other age-related diseases (such as Alzheimer and Parkinson). The decline in tryptophan levels will impair the serotonin and melatonin pathway, which leads to the development of neurological disorders such as depression, cognitive impairment, sleep disorder, Alzheimer, and Parkinson's [49]. Moreover, a decrease in tryptophan levels will also affect protein synthesis leading to weight loss and muscular atrophy [50]. Some of the comorbidities that have been associated with severe COVID-19 are aging, diabetes, hypertension, chronic lung disease, cancer, and HIV. It is well known that the tryptophan-Kyn pathway is activated in the abovementioned conditions [51–55].

Inhibiting Trp-Kyn and/or AhR signaling may represent a novel therapeutic approach for preventing COVID-19-dependent musculoskeletal health and other age-related diseases. There are several Trp-Kyn/Ahr inhibitors that are undergoing clinical trials for various diseased conditions [56]. Currently, indoximod (IDO inhibitor), epacadostat (IDO inhibitor), and IK175 (Ahr inhibitor) are being used for inhibiting Trp-Kyn-Ahr signaling [26].

## 4. Conclusion

**Figure 3:**
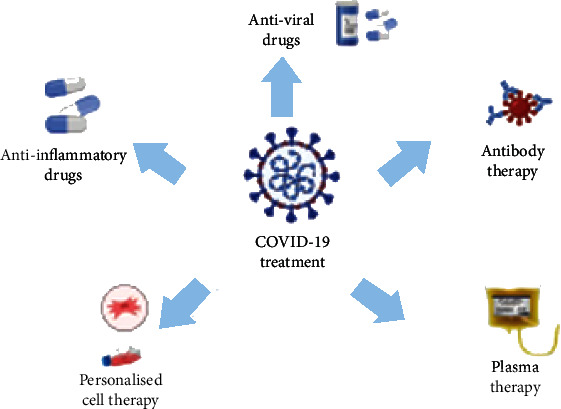
Illustration of various strategies used for COVID-19 treatment. (Figure is created by using http://BioRinder.com.)

Current studies regarding the activation of the IDO-Kyn-AhR pathway in COVID-19 patients have opened up a new frontier for the scientific research community. Based on the available literature, it seems inevitable that activation of the IDO-Kyn-AhR pathway in COVID-19 patients should lead to bone and muscle loss, inducing significant musculoskeletal damage. However, there is currently advancement in COVID-19 therapies (Figure 3), but no strategies are available to address musculoskeletal-related issues. Given that the IDO-Kyn-AhR pathway is activated in COVID-19 patients, the use of inhibitors of IDO and/or AhR might be beneficial to reduce or prevent bone and muscle loss in this disease. IDO1 inhibitors (such as indoximod) and AhR inhibitors (e.g., IK 175) may help prevent bone and muscle loss. Some of these inhibitors are currently in clinical trials to treat several cancers and related complications. However, we suggest the necessity of conducting detailed clinical studies to design therapeutic strategies using these inhibitors to prevent bone and muscle loss in COVID-19 patients. The above-discussed literature is based on old variants of COVID-19. It will be interesting to know how delta and other recent variants circulating in the population will affect the IDO-Kyn-AhR pathway.

## Data Availability

The data supporting this review are from previously reported studies and datasets, which have been cited.
